# A Spatial Covariance ^123^5IA-85380 SPECT Study of α4β2 Nicotinic Receptors in Dementia with Lewy Bodies

**DOI:** 10.1192/bjo.2025.10131

**Published:** 2025-06-20

**Authors:** Judith Harrison, Sean Colloby, John O’Brien, John Paul Taylor

**Affiliations:** ^1^Translational and Clinical Research Institute, Faculty of Medical Sciences, Newcastle University, Newcastle, United Kingdom; ^2^Northumbria NHS Healthcare Foundation Trust, Newcastle, United Kingdom; ^3^Department of Psychiatry, University of Cambridge, Cambridge, United Kingdom; ^4^Central Newcastle Tyne and Wear (CNTW) MHS Foundation Trust, Newcastle, United Kingdom

## Abstract

**Aims:** Cholinergic dysfunction is key in dementia with Lewy bodies (DLB), and likely to influence the cognitive and psychiatric symptoms of this condition. However, patterns of spatial covariance in DLB in terms of nicotinic acetylcholine receptors (nAChRs) is unknown. In this study we used ^123^I-5-iodo-3-[2(S)-2-azetidinylmethoxy] pyridine (^123^5IA-85380) SPECT (α4β2 nAChR assessment) to investigate the covariance patterns in DLB and their associations with cognition.

**Methods:** Fifteen DLB and 16 healthy controls underwent ^123^5IA-85380 and rCBF (^99m^Tc-exametazime) SPECT scanning. We applied voxel principal components (PC) analysis, generating a series of PC images representing common intercorrelated voxels across subjects. Linear regression generated specific α4β2 nicotinic and rCBF covariance patterns that contrasted DLB from controls.

**Results:** A α4β2 pattern that distinguished patients from controls (F_1,29_ = 165.1, p<0.001), showed relative decreased uptake in bilateral temporal pole, inferior frontal, amygdala, olfactory cortex, insula, anterior/mid cingulate and putamen, as well as relative preserved/increased uptake in sensorimotor, fusiform and occipital lobe, implicating regions in a nicotinic receptor expression sense, within limbic, salience, default mode, olfactory, sensorimotor and visual networks. We then successfully derived from patients, α4β2 nicotinic receptor patterns that correlated with CAMCOG_total_ (r=−0.52, p=0.04), MMSE (r=−0.68, p=0.01) and CAMCOG_memory_ (r=−0.70, p=0.01), demonstrating a common ‘cognitive’ topography of relative decreased binding in lateral/medial prefrontal, lateral temporal, inferior parietal and thalamus along with relative preserved/increased binding in cingulate, insula, occipital and medial temporal regions, structures representing a range of networks supporting executive, language, social cognition, attention and sensory functions.

**Conclusion:** In conclusion, disease and cognitive related patterns of cholinergic α4β2 nicotinic receptor binding were apparent in DLB and could inform future therapeutic targets of these receptors in this condition.

